# Histone deacetylase OsHDA716 chilling out with OsbZIP46: antagonistically regulating cold stress tolerance in rice

**DOI:** 10.1093/plcell/koae025

**Published:** 2024-01-24

**Authors:** Nitin Uttam Kamble

**Affiliations:** Assistant Features Editor, The Plant Cell, American Society of Plant Biologists; Biochemistry and Metabolism Department, John Innes Centre, Norwich Research Park, NR4 7UH, Norwich, UK

Plants do not have the luxury of having heated rooms during cold winters as we do. Therefore, to enhance plant cold tolerance and boost agricultural productivity, it is imperative to fully understand how cold perception is signaled inside the cells to activate mechanisms that protect plants from cold damage. It is well studied that a transcriptional cascade involving C-REPEAT BINDING FACTOR/DEHYDRATION-RESPONSIVE ELEMENT-BINDING PROTEIN 1 (CBF/DREB1) induces rapid changes in intracellular calcium levels upon cold stress (Yuan et al. 2018). However, epigenetic mechanisms involved in cold signal transduction are largely unknown, even though epigenetic regulation of gene expression has an essential role in plant adaptation to various environmental changes. Indeed, previous studies demonstrated that histone acetylation/deacetylation is essential to control gene expression in response to abiotic stress (Cheng et al. 2018). Histone deacetylases (HDACs) cause chromatin compaction through the deacetylation of lysine residues in histones, thereby reducing the accessibility of the transcription machinery to gene promoters (Zheng et al. 2020). However, their potential histone-independent functions in cold stress responses remain unexplored.

In this issue of *The Plant Cell*, **Ying Sun and colleagues** (Sun et al. 2024) provide a thorough analysis of how the histone deacetylase OsHDA716 mediates fine-tuning of cold responses in rice by a histone-independent mechanism. Through gene expression analysis in cold conditions, the authors identified *OsHDA716* as a highly responsive gene to cold temperatures. Functional characterization of rice plants with either loss or overexpression of *OsHDA716* showed that it is a negative regulator of cold stress tolerance because loss-of-function mutants were more resistant to cold, whereas plants overexpressing the deacetylase were more susceptible. The authors then turned to analyze the implication of calcium in this process. They used the scanning ion-selective electrode technique (SIET) and Fluo-4 acetoxymethyl ester as fluorescent indicators of [Ca^2+^]_cyt_ and observed the impact of *OsHDA716* on cold-induced Ca^2+^ influx. The results indicated that the negative regulation of rice chilling tolerance by OsHDA716 is connected to a compromised elevation of Ca^2+^ upon cold induction.

Sun et al. further teased out the molecular mechanism underlying this regulatory process by the identification of the transcription factor OsbZIP46 as a protein interaction partner of OsHDA716 using yeast two-hybrid screening. Protein–protein interaction studies confirmed that OsHDA716 physically interacts with OsbZIP46 in vitro and in vivo (Fig.). Importantly, the expression of *OsbZIP46* increases during chilling stress.

The authors confirmed that OsHDA716 deacetylates the lysines in the DNA-binding domain of OsbZIP46 using deacetylation assays. Hence, in addition to its well-known histone-deacetylase activity, OsHDA716 can modulate cold responses by the deacetylation of OsbZIP46, a non-histone substrate. Using ChIP-qPCR and Electrophoretic Mobility Shift Assay (EMSA), the authors observed that deacetylation of OsbZIP46 affects its capacity to bind to the promoters of the cold-responsive genes *OsDREB1A* and *COLD1*, whereas the acetylated form of OsbZIP46 transcriptionally activates them (Fig.). Additionally, deacetylation by OsHDA716 accelerates OsbZIP46 degradation, further decreasing the expression of *OsDREB1A* and *COLD1* and the capacity of the plant to endure cold. Finally, the authors studied the effect of overexpressing *OsbZIP46* in rice plants, observing an increased tolerance of cold stress. Importantly, *OsbZIP46*-overexpression lines had higher expression levels of *COLD1* and *OsDREB1A* in response to chilling stress.

**Figure.**
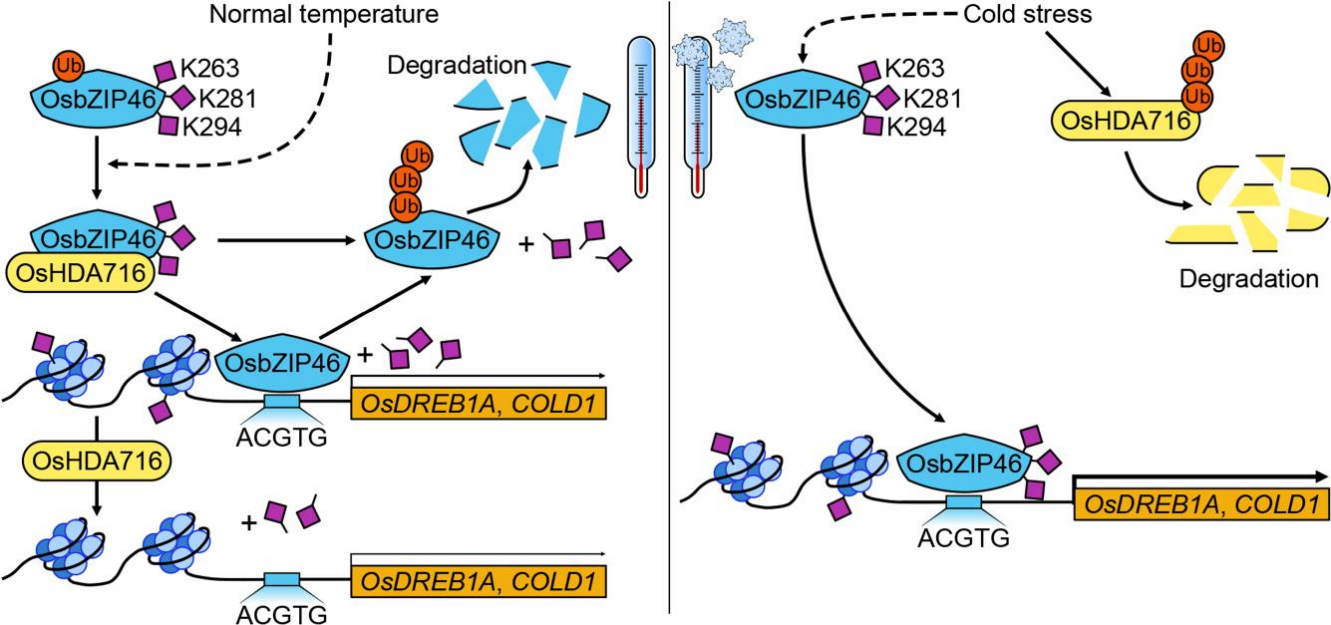
OsHDA716 and bZIP46 antagonistically regulate cold stress tolerance in rice. During cold stress, OsHDA716 degradation leads to acetylated OsbZIP46 accumulation with transactivation of downstream target genes conferring chilling tolerance. However, during control growth conditions, deacetylation mediated by OsHDA716 leads to the OsbZIP46 degradation inhibiting transactivation of downstream target genes. Ac, lysine acetylation. Reprinted from Sun et al. (2024), Figure 10.

Taken together, these findings shed light on how histone deacetylases can repress transcriptional activity by deacetylating non-histone substrates. In particular, OsHDA716 deacetylates the transcription factor OsbZIP46, inhibiting its capacity to activate the transcription of downstream cold responsive genes, fine-tuning cold responses in rice. Further investigation is needed to determine how OsbZIP46 is acetylated in the first place and how OsbZIP46 deacetylation by OsHDA716 in the absence of cold stress reduces protein stability. It is also important to ascertain whether OsbZIP46 transcriptionally regulates OsHDA716 in response to cold stress. Understanding these mechanisms can have significant implications for enhancing cold tolerance in crop plants.
